# Spinal cord injury in the emergency context: review of program outcomes of a spinal cord injury rehabilitation program in Sri Lanka

**DOI:** 10.1186/1752-1505-8-4

**Published:** 2014-03-20

**Authors:** Jo C Armstrong, Brooke E Nichols, Joan M Wilson, Roy A Cosico, Leslie Shanks

**Affiliations:** 1 Médecins Sans Frontières, Operational Centre Amsterdam, Amsterdam, the Netherlands; 2 Department of Viroscience, Erasmus Medical Centre, Rotterdam, the Netherlands

**Keywords:** Spinal cord injury, Rehabilitation, Emergency response, Conflict, Outcomes, Médecins Sans Frontières (MSF), Sri Lanka

## Abstract

**Background:**

The final months of the conflict in Sri Lanka in 2009 resulted in massive displacement of the civilian population and a high volume of orthopedic trauma including spinal cord injury. In response to this need, Médecins Sans Frontières implemented a multidisciplinary rehabilitation program.

**Methods:**

Patients were admitted to the program if they had a spinal cord injury, a stable spine and absence of a high-grade pressure ulcer. All patients were assessed on admission with a standardized functional scale the Spinal Cord Independence Measure II (SCIM) and the American Spinal Injury Association Impairment Scale (ASIA). A multidisciplinary team provided nursing care, physiotherapy, bowel and bladder training, mental health care, and vocational rehabilitation. Patients were discharged from the program when medically stable and able to perform activities of daily living independently or with assistance of a caregiver. The primary outcome measures were discharge to the community, and change in SCIM score on discharge. Secondary outcome measures were measured at 6-12 weeks post-discharge, and included SCIM score and presence of complications (pressure ulcers, urinary tract infections and bowel problems).

**Results:**

89 patients were admitted. The majority of injuries were to the thoracic region or higher (89%). The injuries were classified as ASIA grade A in 37 (43%), grade B in 17(20%), grade C in 15 (17%) and grade D in 17(20%). 83.2% met the criteria for discharge, with a further 7.9% patients requiring transfer to hospital for surgical care of pressure ulcers. There was a significant change in SCIM score from 55 on admission to 71 on discharge (p < 0.01). 79.8% and 66.7% achieved a clinically significant and substantially significant SCIM score improvement, respectively. Amongst those with follow up data, there was a reduction in post spinal cord injury complications from those experienced either at or during admission. A further 79% of SCIM scores were stable or improved compared to the score on discharge.

****Conclusion**s:**

Provision of effective rehabilitation for spinal cord injury is possible in complex humanitarian emergency situations. A multidisciplinary approach, including psychological support along with partnerships with local and international organizations with specialized expertise, was key to the program’s success.

## Background

Sri Lanka has emerged from a 26-year conflict between the Sri Lankan Army government forces (SLA) and the Liberation Tigers of Tamil Eelam (LTTE). Between January and May 2009 the fighting was concentrated on a constantly shrinking and densely populated area and the numbers of civilian victims increased sharply. Around 330,000 civilians were displaced to a narrow conflict zone in the Northeast. After the LTTE’s defeat by the SLA in May 2009, the army escorted the survivors to government-controlled camps in Vavuniya, central Sri Lanka. The international humanitarian organization, Médecins Sans Frontières (MSF) was present in the area to provide emergency medical assistance to the injured. Between February and June, MSF treated almost 4000 war wounded [1]. A cohort of 60 patients with spinal cord injury (SCI) was identified from amongst this group in the first months of intervention. These patients were admitted to the MSF hospital and received general nursing care and physiotherapy services similar to other orthopedic patients. However this cohort of patients proved a dilemma for MSF teams as there were no local specialized rehabilitation facilities available and the alternative of discharge back to the internally displaced person (IDP) camps would have been catastrophic for this highly vulnerable patient group. MSF, however, had little experience in providing the specialized rehabilitation services required and was concerned about its ability to support complex needs such as bladder training and skin care. A related concern was the likelihood of discharging SCI patients to the community, as MSF’s presence in the region would be limited to the immediate post-emergency phase. After considerable internal debate, MSF decided to go ahead with implementing a spinal cord injury rehabilitation program. One of the key arguments MSF used to support the program was the evidence that without rehabilitation, the odds of survival were poor [2-4]. This paper describes the intervention, analyzes the results, and presents recommendation for future interventions.

## Methods

### Setting

The program was located in Vavuniya, close to the large IDP camps. A 30-bed rehabilitation unit was built on the grounds of an existing hospital. A mobility skills area, basketball/volleyball court, and physiotherapy area were constructed on the same site.

### The program

An MSF expatriate team was created consisting of a mental health specialist, nurse, medical doctor, logistician, and physiotherapist. They worked with nursing and medical staff recruited from the Ministry of Health (MoH). The MSF medical coordinator (JW) set up the program with support from MSF headquarters, input from external rehabilitation specialists and a locally based consultant physiotherapist experienced in working with SCI patients (JA). The local consultant provided staff training in wheelchair and seating. An orthopedic surgeon provided consultation when needed. Throughout the program there was limited turn-over of staff with expatriate positions and MoH physicians staying approximately 6 months, and most national staff present for the duration of the program.

External rehabilitation specialists from the UK provided training in bladder and bowel management, wound care and pressure ulcer healing for both staff and patients. The training took place over two sessions for a total of three weeks, with the first session taking place at the start of the program. Handicap International and Motivation Charitable Trust provided assistive devices such as walking aids and all terrain wheelchairs with pressure relief cushions. A team of peer group trainers from the UK visited the program to provide a training course for 10 patients identified as showing aptitude for peer support. A subsequent peer-training workshop was held in the last quarter of 2011 led by these national peer trainers. Pain management was provided using standard MSF protocols: Paracetamol was used as a first line management; Amitriptyline and/or Carbamazepine were prescribed for neuropathic pain.

Trained lay mental health workers provided motivational support and counseling to all patients and their caretakers. This was particularly important as the cohort faced not only the psychological impact of SCI but also traumatic experiences and multiple losses arising from the conflict.

### Admission

Patients with a spinal cord injury were admitted to rehabilitation if they had a stable vertebral spine as determined by X-ray examination. Those with high-grade pressure ulcers were excluded until healing had progressed to a satisfactory stage. Caretaker support from a family member or friend was encouraged but not mandatory given so many people had lost or become separated from relatives in the conflict.

Assessment on admission was made with the American Spinal Injury Association Impairment Scale (ASIA), and Spinal Cord Independence Measure II (SCIM). The ASIA scale is used internationally to determine the neurologic level of impairment for sensation and motor power [5]. The SCIM II is a 16-domain disability scale covering self-care, respiratory and sphincter management and mobility with a possible total of 100 points [6-8]. A higher SCIM score correlates with a higher level of functional independence. Based on this assessment, an individualized program was developed with the patient and caregiver. Multidisciplinary meetings were held on a fortnightly basis to discuss patient progress and adapt care plans if necessary.

### Discharge

Patients were discharged from the program when medically stable and able to perform activities of daily living independently or with assistance of a caregiver. Prior to discharge, the discharge coordinator worked with each individual and their caregiver to develop an educational and/or occupational plan. Many individuals benefited from vocational training provided by local community organizations prior to discharge. Examples of the training provided by these local organizations include cell phone repair, computer courses, computer repair and the making of handicrafts. An auto rickshaw, a popular local means of transport, was adapted on site to allow driver training to take place. Other clients were facilitated to apply for educational bursaries, training positions or to renew their professional registrations.

### Follow-up post discharge

Follow-up was planned at 6–12 weeks post discharge to assess progress and provide support. Constraints, due to lack of transport and absence of Ministry of Defense travel clearance meant that a number of individuals were followed up by telephone. Information gathered on follow-up included the presence of complications such as pressure ulcers, urinary tract infections (UTI), bowel problems, pain, and psychological problems. Where possible, SCIM scores were repeated.

### Outcome measures

The primary outcome measures used were successful discharge to the community and absolute change in SCIM score between admission and discharge. A positive outcome in terms of change in SCIM score was quantified as either a small clinical improvement (>4.5 points) or a substantial improvement (>10.0 points) [9]. Secondary outcomes measures were SCIM score on follow-up at 6-12 weeks and presence of complications at 6-12 weeks follow-up.

### Study design and statistical analysis

The design is a retrospective analysis of routinely collected programmatic data. All patients admitted to the spinal cord rehabilitation program were included in the analysis.

To identify all possible variables associated with SCIM-score improvement, all relevant variables were analyzed using one-way ANOVA analyses (Table 1). Variables with a positive, significant association (p < 0.05) with a SCIM-score improvement and all potentially relevant confounders were used in our final model using ANCOVA analyses. Measured variables considered as potential confounders include: age, sex, number of days in rehabilitation, ASIA score, time from injury to hospital admission, time from injury to rehabilitation, and injury level. A p-value of <0.05 in our ANOVA & ANCOVA analyses signified a significant improvement in the respective outcome variable between admission and discharge (SCIM Score), and between admission and after follow-up (pressure ulcers, UTIs, bowel problems, pain and psychological problems) (Table 2). The data was analyzed using the statistical package SAS 9.2 (SAS Institute Inc., Cary, NC)).

**Table 1:** Sri Lankan spinal cord injury rehabilitation cohort demographics & injury characteristics

***Demographics***	**Total cohort (%) (n = 89)**	SCIM score gain* (SD) (n = 84)	p-value**
*Age*			0.36
<21	16 (18%)	14.8 (11.0)	
21-30	38 (43%)	14.8 (11.0)	
31-35	14 (16%)	12.4 (8.1)	
36+	21 (23%)	19.3 (15.4)	
*Sex*			0.14
Female (N(%))	26 (29%)	18.5 (11.7)	
Male	63 (71%)	14.3 (11.3)	
*Education*			0.76
Sub-O levels	62 (70%)	16.1 (10.6)	
O levels	16 (18%)	13.9 (15.2)	
A levels	11 (12%)	14.4 (12.1)	
*Occupation*			0.4
Manual	33 (50%)	16.7 (13.6)	
Office	15 (23%)	15.3 (10.4)	
Education	18 (27%)	11.9 (9.0)	
***Injury characteristics***			
*SCIM score on admission*			<0.01
<40	12 (13%)	15.3 (12.2)	
40-55	30 (34%)	22.6 (13.4)	
56-70	32 (36%)	13.4 (8.0)	
> 70	15 (17%)	7.1 (6.7)	
*ASIA score*			0.15
A	37 (43%)	15.4 (8.5)	
B	17 (20%)	13.6 (10.6)	
C	15 (17%)	11.8 (12.6)	
D	17 (20%)	20.7 (16.0)	
*Injury level*			
Cervical	13 (15%)	12.0 (8.3)	0.35
Thoracic	64 (74%)	16.8 (12.0)	
Lumbar***	9 (10%)	13.0 (12.4)	
*Length of stay, rehabilitation*			0.31
<127	22 (25%)	19.6 (12.6)	
127-210	23 (26%)	15.7 (11.1)	
211-290	21 (24%)	15.4 (10.7)	
>290	22 (25%)	12.5 (12.1)	
Duration of stay of readmission, mean (SD)	236 (137)	15.5 (11.7)	0.36
*Time from Injury to hospital admission*			<0.01
<6 months	28 (31%)	22.8 (13.2)	
>6 months	61 (69%)	12.0 (9.1)	
*Time from hospital admission to admission to rehabilitation*			0.92
<6 months	59 (67%)	15.1 (10.8)	
>6 months	29 (33%)	15.3 (12.5)	
*Time from Injury to admission to rehabilitation*			<0.01
<6 months	8 (9%)	30.8 (14.5)	
>6 months	79 (91%)	13.5 (9.6)	
*Family or friend support during care*			0.92
Yes	36 (40%)	15.3 (9.9)	
No	53 (60%)	15.6 (12.8)	

*Difference in SCIM score between admission and discharge.

**To identify all possible variables associated with SCIM-score improvement, all variables analyzed using one-way ANOVA analyses.

***Includes one patient with a sacral injury.

**Table 2:** Sri Lankan spinal cord injury rehabilitation cohort outcome indicators: SCIM scores and complications

	*On admission*	*At discharge*			Fully adjusted p-value*
SCIM score - *mean, SD; n = 84*	55 (18)	71 (19)			*<0.01*
***Complications***	*On admission/during stay*	*On follow up*			
		Resolved	Persistent**	New***	*Unadjusted p-value*
Pressure ulcers*- N (%); n = 51*	29 (57%)	21 (41%)	8 (16%)	3 (6%)	*<0.01*
Urinary tract infection*- N (%); n = 51, n = 49†*	23 (45%)	18 (37%)	4 (8%)	1 (2%)	*<0.01*
Bowel problems*- N (%); n = 50*	34 (68%)	28 (56%)	6 (12%)	0 (0%)	*<0.01*
Pain*- N (%); n = 51*	38 (75%)	8 (16%)	30 (59%)	2 (4%)	*0.92*
Psychological problems*- N (%); n = 25, n = 24†*	23 (92%)	15 (63%)	7 (29%)	2 (8%)	*0.86*

*Based on ANCOVA analyses and adjusted for variables associated with a significant SCIM-score improvement, and all potentially relevant confounders: age, sex, number of days in rehabilitation, ASIA score, time from injury to hospital admission, time from injury to rehabilitation, and injury level.

**Persistent refers to those who had a complication during stay and continued to have the complication after discharge.

***New refers to those who did not have the complication during stay, but developed it after returning home.

†Number of patients on follow-up, if different.

### Missing data

SCIM-scores were available for 84 patients (94%), information on pressure ulcers, UTIs, and pain were available for 51 patients (57%), on bowel problems 50 patients (56%), and on psychological problems for 25 patients (28%). Statistical analyses on the respective outcome variables were conducted with the available data. Additionally, we determined whether patients for whom we had data differed from those we did not with respect to age, sex, SCIM-score on admission, ASIA grade or injury level.

### Ethics statement

This study met the standards set by the MSF Ethics Review Board for retrospective analysis of routinely collected programmatic data. The database was anonymized prior to analysis.

## Results

### Patient description

Between November 2009 and December 2010, 89 patients were admitted to the rehabilitation program. Patients had a mean age of 30 years on admission (standard deviation (SD) 11.2). The majority was male (n = 63, 71%). Injury levels ranged from C2 to S1, with most having an injury at the thoracic vertebral level (n = 64, 74%). The injuries were classified as ASIA grade A in 37 (43%), grade B in 17(20%), grade C in 15 (17%) and grade D in 17(20%). The mean SCIM score on admission was 55 (SD 18.3). Most injuries occurred greater than six months prior to being admitted to the hospital (n = 57, 69%). Very few patients were admitted to the rehabilitation program within six months of injury (n = 8, 9%) (Table 1).

### Outcomes

74 (83.2%) patients met the criteria for discharge. The average length of stay for discharged patients was 223 days (SD 117). Eight (9.0%) defaulted at a median of 100 days. Seven (7.9%) required transfer to hospital for surgical intervention for pressure ulcers. One of these patients was transferred to another hospital for chronic care; the remaining six individuals were all eventually discharged to the community. Seven (7.9%) individuals required short-term re-admission to rehabilitation due to medical complications or deterioration in SCIM score after a median of 180 days (SD 100).

84.5% (n = 71, SD = 19) of patients had an increase in SCIM score, with a further 15.5% remaining stable, showing that most patients’ functional abilities improved with only a small minority showing no improvement. 79.8% improved by >4.5 points, representing a clinically significant change, while 66.7% improved by >10 points representing a substantial clinically significant change. SCIM scores of the total cohort improved from 55 (SD 18) on admission to 71 (SD 19) on discharge (adjusted for age, sex, duration of rehabilitation, ASIA grade, time from injury to hospital admission, time from injury to rehabilitation admission, and injury level *p* = <0.01). On univariate analysis, three of 13 variables were associated with an increase in SCIM score: SCIM score on admission, time from injury to hospital admission, and time from injury to rehabilitation admission (Table 1). Patients with lower SCIM scores on admission had larger SCIM score gains, those with a SCIM score of <40 and 40-55 on admission gained an average of 15.3 (SD 12.2) and 22.6 (SD13.4) points. Those with higher SCIM scores on admission had relatively lower SCIM score increases; those with a SCIM score of 56-70 and 70+ on admission gained an average of 13.4 (SD 8.0) and 7.1 (SD 6.7), respectively. Patients admitted to the hospital within six months of injury had larger gains in SCIM score compared to those who were not, 22.8 (SD 13.2) vs. 12.0 (SD 9.1), respectively. The patients who were admitted to the rehabilitation program within six months of their injury had the largest SCIM score increases of all subgroups analyzed. These individuals had an average gain in SCIM score of 30.8 (SD 14.5) points, compared with the individuals who were admitted to rehabilitation greater than six months after injury with an increase of 13.5 (SD 9.6) points.

Of the 28 (31.5%) patients for whom follow-up SCIM scores were available, 3 (11%) improved between discharge and 6-12 weeks follow-up, 19 (68%) were stable, and 6 (21%) had a decline.

### Complications

Information on complications was available for 51 (57.3%) patients. A large number of patients had pressure ulcers either on or during admission to rehabilitation (29 of 51 patients) (Table 2). Of these 29 patients, 21 had no recurrence on follow-up, 8 persisted, and 3 patients with no history of ulcer developed a new pressure ulcer in the community (*p* = <0.01). During rehabilitation, 23 patients (45%) had a UTI. At follow-up in the community, 18 of the 23 patients had no further UTIs, 4 continued to have this complication at least once, and 1 additional person developed a new UTI (*p* = <0.01) (2 were lost to follow-up). 34 patients (68%) had bowel problems during their hospital stay. At follow-up, 28 of these had resolved, 6 persisted, while no additional patients developed bowel problems (*p* = <0.01). The majority (38 of 51, 75%) complained of pain on or during admission. At follow-up, pain persisted for 30 patients (59%), and was a new complaint for 2 (4%). Information on presence of psychological problems is available for 25 patients, and 23 of these (92%) reported the problem on or during admission. There was no statistically significant reduction in pain or psychological problems at follow-up (*p = 0.92, p = 0.86* respectively).

## Discussion

This report describes standardized treatment outcomes of a cohort of SCI cases undergoing rehabilitation in a humanitarian setting. All but one patient were successfully discharged to the community, and almost 80% achieved a clinically significant improvement in SCIM score. Some of the largest improvements in SCIM score were seen among patients who began rehabilitation within six months of injury, illustrating the importance of initiating rehabilitation as soon as possible. However even amongst the group admitted to rehabilitation more than 6 months after the injury, substantial clinical improvement was found (mean SCIM increase 13.5). This is notable given the fact that the individuals injured in the conflict had little or no access to medical care at the time of their injury and often for months thereafter.

Comparison of our functional outcomes with those in Western settings is challenging due to lack of a standardized reporting conventions for functional improvement. We have referenced recent work by Scivoletti *et al.* that offers a possible solution to the gap in reporting outcomes [9]. They propose a methodology using a later version of the SCIM score, namely version III. In their database of 255 acute and sub-acute SCI patients treated in their hospital in Italy, 94% and 91% showed clinical and substantial improvement respectively. Our results are lower than this, however the patient groups differ particularly due to the fact that none of our patients presented acutely due to the delay in seeking care imposed by the conflict. A further limitation in comparing outcomes is that our dataset did not include the details of the subgroup scoring.

Several authors describe health complications of SCI populations in the humanitarian emergency context [10-13]. While comparisons are difficult due to variation in the cohort composition and in the methodology, many of the reports have similarly high rates of SCI complications as observed in our population (See Table 3). Pressure ulcers proved challenging as evidenced by the seven individuals who had pressure ulcers that worsened to the point of needing surgical intervention; four of these tested positive for methicillin-resistant Staphylococcus aureus (MRSA) and required specialized antibiotics. In addition, three individuals developed pressure sores for the first time after discharge. Overall the incidence of the five categories of complications improved at follow-up as compared to those reported at or during admission. This change was found to be statistically significant for pressure ulcers, UTIs and bowel problems.

**Table 3:** Review of post SCI complications in other settings

**Setting**	**Cohort description**	**Complications**
**Iran: 2003 earthquake**[10]	61 SCI patients were surveyed eight months after the Bam earthquake.	• Pressure ulcers: 35%
		• UTI: 9%
		• Bowel problems: 46.3%
		• Pain: 96%
**Pakistan: 2005 earthquake**[11]	194 patients hospitalized with SCI over a two-month period immediately following the earthquake were assessed. ASIA classification of injuries was 46% in A, 4% (8) in B, 11% (21) in C, 9% (18) in D, and 14% (27) in E.	• Pressure ulcers: 20%
		• UTI: 100% (majority of patients had indwelling catheters)
		• Bowel problems: 15%
**Afghanistan: war trauma**[12]	A cross sectional survey of 311 traumatic SCI patients in Kabul and Herat who had received rehabilitation at International Committee of the Red Cross (ICRC) supported facilities is reported. The median age of the injury was seven years. Level of injury was thoracic 47%, lumbar 46%, cervical 7% and sacral 1%.	• Pressure ulcers: 32%
		• UTI: 57%
		• Pain: 74%
		• Joint contractures: 43%
		• Negative feelings: 59%
**China: 2008 earthquake**[13]	51 SCI patients were admitted to rehabilitation in three hospitals. 94% were admitted within four months of injury. The ASIA classification on admission showed 22 in A, 8 in B, 15 in C, 5 in D and 1 not classified.	• Pressure ulcer: 44%
		• UTI: 33%
		• Neurogenic bladder: 59%
		• Neurogenic bowel: 61%
		• Pain: 33%

The average LOS (223 days) is longer than typical rehabilitation times in North America and Europe [14]. However this was exacerbated by the uncertain conditions for discharge in both the IDP camps and in the home communities that had been destroyed by war.

Our program was expensive, costing €664,818 in total. 67% of the budget represented staff costs. Looking forward, it is important to identify ways in which to reduce both staffing costs and LOS without reducing quality of care to make this type of intervention more financially feasible.

Key to the success of the program was the multidisciplinary approach, which included psychological support and partnerships with specialized organizations. Some of the specific expertise came from outside Sri Lanka, but as much as possible patients were linked with local organizations such as the Vanni Rehabilitation Organisation for the Differently Abled (VAROD) and the Sri Lankan Society for Prosthetics and Orthotics (SLSPO) in order to ensure continued support after MSF left. A particular challenge to the program came from travel restrictions that limited community support post-discharge, as most patients were being discharged to communities distant from the rehabilitation centre. The training of expert patients to provide peer support successfully compensated this limitation.

A limitation is the fact that the analysis was done on routinely collected programmatic data, which may have had an impact on data quality and resulted in missing data. This missing data may have impacted the results of our analyses. The patients on whom information regarding pressure ulcers, UTIs, bowel problems and pain was available were not statistically different in age, sex, SCIM-score on admission, ASIA grade or injury level than those for whom data was missing. However the patients for whom data on these variables was available did have a significantly higher SCIM-score at discharge compared to those who we did not have information on (p = 0.04). Therefore, it is possible that the impact of the rehabilitation program on reducing the complications of pressure ulcers, UTIs and bowel problems may have been overestimated. The overall outcome measure of improvement in SCIM-scores between admission and discharge is not subject to this limitation, as just five patients had missing SCIM-score information. These five patients did not differ significantly on the variables described above.

## Conclusions

Our results suggest that such a program is possible under difficult circumstances and encourages other non-specialized organizations to consider offering SCI rehabilitation as part of their emergency response where large numbers of SCI are sustained. We were able to demonstrate substantial clinical improvement as measured by standardized scores and successful discharge to the community, despite the fact that most patients entered rehabilitation more than six months from their injury and had delayed access to medical care at the time of the injury. Further research is needed to determine how to streamline the approach in order to reduce both length of stay and high costs linked to staffing.

## Abbreviations

SCIM: Spinal cord independence measure; SLA: Sri Lankan army; LTTE: Liberation Tigers of Tamil Eelam; MSF: Médecins Sans Frontières; SCI: Spinal cord injury; IDP: Internally displaced person; ASIA: American Spinal Injury Association Impairment Scale; UTI: Urinary tract infection; LOS: Length of stay; MRSA: Methicillin-resistant staphylococcus aureus.

## Competing interests

JA was employed by MSF Holland as a consultant during the implementation of the program.

## Authors’ contributions

LS participated in the study design and helped to draft the manuscript, BN performed the statistical analysis and helped to draft the manuscript, JA participated in the study design and helped to draft the manuscript, JW and RC were responsible for the study implementation and contributed to editing the manuscript. All authors read and approved the final manuscript.
